# Substance Addiction Among Medical Residents in The City of Sousse : Prevalence and Associated Factors

**DOI:** 10.1192/j.eurpsy.2026.10864

**Published:** 2026-07-31

**Authors:** G. Ltaief, A. Karmous, O. Ajmi, J. Nakhli

**Affiliations:** Psychiatry Department, Farhat Hached Hospital, Sousse, Tunisia

## Abstract

**Introduction:**

Substance addiction is a major public health issue affecting all segments of the population, including healthcare professionals. Among them, medical residents are a particularly vulnerable group due to their demanding working conditions, which combine intense mental stress, long hours and frequent exposure to emotionally stressful situations.

**Objectives:**

This study aimed to assess the prevalence of addictions (tobacco, alcohol, cannabis) among medical residents in the city of Sousse (Tunisia), identify associated factors and compare specialities.

**Methods:**

A cross-sectional study was conducted over three months, from January 2025 to March 2025, through an online survey. Medical residents who were working at Farhat Hached and Sahloul hospitals of Sousse (Tunisia), during the study period were included. Socio-demographic data, medical history, and lifestyle habits were collected, supplemented by three validated tools including the Alcohol Use Disorders Identification Test, the Cannabis Abuse Screening Test and the Fagerström Test.

**Results:**

A total of 210 medical residents fully responded to the survey. The mean age was 27.8 years, with 59% being women. Among participants, 23.8% were tobacco smokers, 33.3% alcohol consumers, and 15.2% cannabis users. Tests revealed low nicotine dependence in 58% of smokers, risky alcohol consumption in 50% of drinkers and problematic cannabis use (43.8%). Surgical residents showed higher rates of addiction for the three substances being studied compared to residents of medical specialities (tobacco: 39.4% vs 15.8%; alcohol: 53.5% vs 23%; cannabis: 35.2% vs 5%; p<0.001).

**Conclusions:**

This study highlights an alarming prevalence of addictions among residents, particularly in surgery, suggesting a link to professional stress. It emphasizes the urgent need for preventive measures involving systematic screening, psychological support, and improved working conditions, especially in high-risk specialities.

**Disclosure of Interest:**

None Declared

